# Machine Learning and Its Applications to Biology

**DOI:** 10.1371/journal.pcbi.0030116

**Published:** 2007-06-29

**Authors:** Adi L Tarca, Vincent J Carey, Xue-wen Chen, Roberto Romero, Sorin Drăghici

**Affiliations:** Whitehead Institute, United States of America

## Introduction

The term *machine learning* refers to a set of topics dealing with the creation and evaluation of algorithms that facilitate pattern recognition, classification, and prediction, based on models derived from existing data. Two facets of mechanization should be acknowledged when considering machine learning in broad terms. Firstly, it is intended that the classification and prediction tasks can be accomplished by a suitably programmed computing machine. That is, the product of machine learning is a classifier that can be feasibly used on available hardware. Secondly, it is intended that the creation of the classifier should itself be highly mechanized, and should not involve too much human input. This second facet is inevitably vague, but the basic objective is that the use of automatic algorithm construction methods can minimize the possibility that human biases could affect the selection and performance of the algorithm. Both the creation of the algorithm and its operation to classify objects or predict events are to be based on concrete, observable data.

The history of relations between biology and the field of machine learning is long and complex. An early technique [1] for machine learning called the perceptron constituted an attempt to model actual neuronal behavior, and the field of artificial neural network (ANN) design emerged from this attempt. Early work on the analysis of translation initiation sequences [2] employed the perceptron to define criteria for start sites in Escherichia coli. Further artificial neural network architectures such as the adaptive resonance theory (ART) [3] and neocognitron [4] were inspired from the organization of the visual nervous system. In the intervening years, the flexibility of machine learning techniques has grown along with mathematical frameworks for measuring their reliability, and it is natural to hope that machine learning methods will improve the efficiency of discovery and understanding in the mounting volume and complexity of biological data.

This tutorial is structured in four main components. Firstly, a brief section reviews definitions and mathematical prerequisites. Secondly, the field of supervised learning is described. Thirdly, methods of unsupervised learning are reviewed. Finally, a section reviews methods and examples as implemented in the open source data analysis and visualization language R (http://www.r-project.org).

## Main Concepts and Definitions

Two main paradigms exist in the field of machine learning: *supervised* and *unsupervised* learning. Both have potential applications in biology.

In supervised learning, objects in a given collection are classified using a set of attributes, or features. The result of the classification process is a set of rules that prescribe assignments of objects to classes based solely on values of features. In a biological context, examples of *object*-to-*class* mappings are tissue gene expression profiles to disease group, and protein sequences to their secondary structures. The features in these examples are the expression levels of individual genes measured in the tissue samples and the presence/absence of a given amino acid symbol at a given position in the protein sequence, respectively. The goal in supervised learning is to design a system able to accurately predict the class membership of new objects based on the available features. Besides predicting a categorical characteristic such as class label, (similar to classical *discriminant analysis*), supervised techniques can be applied as well to predict a continuous characteristic of the objects (similar to *regression analysis*). In any application of supervised learning, it would be useful for the classification algorithm to return a value of “doubt” (indicating that it is not clear which one of several possible classes the object should be assigned to) or “outlier” (indicating that the object is so unlike any previously observed object that the suitability of any decision on class membership is questionable).

In contrast to the supervised framework, in unsupervised learning, no predefined class labels are available for the objects under study. In this case, the goal is to explore the data and discover similarities between objects. Similarities are used to define groups of objects, referred to as *clusters*. In other words, unsupervised learning is intended to unveil natural groupings in the data. Thus, the two paradigms may informally be contrasted as follows: in supervised learning, the data come with class labels, and we learn how to associate labeled data with classes; in unsupervised learning, all the data are unlabeled, and the learning procedure consists of both defining the labels and associating objects with them.

In some applications, such as protein structure classification, only a few labeled samples (protein sequences with known structure class) are available, while many other samples (sequences) with unknown class are available as well. In such cases, *semi-supervised* techniques can be applied to obtain a better classifier than could be obtained if only the labeled samples were used [5]. This is possible, for instance, by making the “cluster assumption,” i.e., that class labels can be reliably transferred from labeled to unlabeled objects that are “nearby” in feature space.

Life science applications of unsupervised and/or supervised machine learning techniques abound in the literature. For instance, gene expression data was successfully used to classify patients in different clinical groups and to identify new disease groups [6–9], while genetic code allowed prediction of the protein secondary structure [10]. Continuous variable prediction with machine learning algorithms was used to estimate bias in cDNA microarray data [11].

To support precise characterization of both supervised and unsupervised machine learning methods, we have adopted certain mathematical notations and concepts. In the next sections, we employ vector notation (**x** denotes an ordered *p*-tuple of numbers for some integer *p*), matrix notation (X denotes a rectangular array of numbers, where *x_ij_* will denote the number in the *i*th row and *j*th column of X), conditional probability densities, and sufficient matrix algebra to define the multivariate normal density. Necessary formal background in algebra and probability can be found elsewhere [12].

## Supervised Learning

### 

#### General concepts.

Let us consider the general case in which we want to classify a collection of objects *i* = 1, . . ., *n* into *K* predefined classes. For instance, if one wants to distinguish between different types of tumors based on gene expression values, then *K* would represent the number of known existing tumor types. Without loss of generality, data on features can be organized in an *n* × *p* matrix X = (*x_ij_*), where *x_ij_* represents the measured value of the variable (feature) *j* in the object (sample) *i*. Every row of the matrix X is therefore a vector **x** *_i_* with *p* features to which a class label *y_i_* is associated, *y* = 1,2,. . .,*c*,. . .,*K*. In such multiclass classification problems, a classifier *C*(**x**) may be viewed as a collection of *K* discriminant functions *g_c_*(**x**) such that the object with feature vector **x** will be assigned to the class *c* for which *g_c_*(**x**) is maximized over the class labels *c* ∈ {1,. . .,*K*}. The feature space *X* is thus partitioned by the classifier *C*(**x**) into *K* disjoint subsets.

There are two main approaches to the identification of the discriminant functions *g_c_*(**x**) [13]. The first assumes knowledge of the underlying class-conditional probability density functions (the probability density function of **x** for a given class) and assigns *g_c_*(**x**) = *f*(*p*(**x** | *y* = *c*)), where *f* is a monotonic increasing function, for example the logarithmic function. Intuitively, the resulting classifier will classify an object **x** in the class in which it has the highest membership probability. In practice, *p*(**x** | *y* = *c*) is unknown, and therefore needs to be estimated from a set of correctly classified samples named *training* or *design* set. Parametric and nonparametric methods for density estimation can be used for this end. From the parametric category, we will discuss linear and quadratic discriminants, while from the nonparametric one, we will describe the *k*-nearest neighbor (*k*-NN) decision rule. The second approach is to use data to estimate the class boundaries directly, without explicit calculation of the probability density functions. Examples of algorithms in this category include decision trees, neural networks, and support vector machines (SVM).

#### Error estimation.

Suppose the classifier *C*(**x**) was trained to classify input vectors **x** into two distinct classes, 1 and 2. The classification result on a collection of input objects **x** *_i_*, *i* = 1,. . .,*n* can be summarized in a *confusion matrix*. The confusion matrix contrasts the predicted class labels of the objects 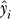 with the true (given) class labels *y_i_*. An example confusion matrix computed for 100 objects is:

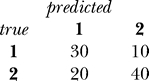



The *error rate* (*Err*) of the classifier is defined as the average number of misclassified samples, i.e., the sum of off-diagonal elements of the confusion matrix, divided by the total number of objects. In the example above, *Err* = (10 + 20) / 100 = 30%. Conversely, the *accuracy* of the classifier can be defined as *Acc* = 1 − *Err* = 70% and represents the fraction of samples successfully classified.

The goal behind developing classification models is to use them to predict the class membership of *new samples*. If the data used to build the classifier is also used to compute the error rate, then the resulting error estimate, called the *resubstitution* estimate, will be optimistically biased [14]. A better way to assess the error is the *hold-out* procedure in which one splits the data into two equal parts. The first half is used to train the classifier (the *training set*), while the remaining half is used to assess the error (the *test set*). With biological data, this approach is rarely feasible due to the paucity of the data. A more appropriate alternative is the *leave-one-out* cross-validation method (LOO) which trains the classifier *n* times on (*n* − 1) samples, omitting each observation in turn for testing the classifier. The *n* test results obtained in this way can be arranged into a confusion matrix, and *Err* estimated by the proportion of off-diagonal elements. Although the estimate of the error obtained with the leave-one-out procedure gives low bias, it may show high variance [15]. A good tradeoff between bias and variance may be obtained by using *N-fold cross-validation* in which the dataset is split into (*n* − *m*) training points and *m* test points (*N* = *n/m*). Using multiple resampling, one can obtain a mean, as well as a standard deviation, for the classifier error.

#### Types of classifiers.

*Quadratic and linear discriminants.* A standard classification approach, applicable when the features are continuous variables (e.g., gene expression data), assumes that for each class *c*, **x** follows a multivariate normal distribution *N*(**m** *_c_*,Σ*_c_*) having the mean **m** *_c_* and covariance matrix Σ*_c_.* The covariance matrix Σ is square with dimension *p* × *p*. The element *i*,*j* of this matrix is the covariance between the variables *i* and *j*.

Using the multivariate-normal probability density function and replacing the true class means and covariance matrices with sample-derived estimates (**m** *_c_* and 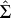
_c_, respectively), the discriminant function for each class can be computed as:

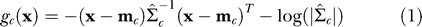
where

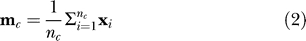
and

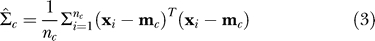



The discriminant functions are monotonically related to the densities *p*(**x** | *y* = *c*), yielding higher values for larger densities. The values of the discriminant functions will differ from one class to another only on the basis of the estimates of the class mean and covariance matrix. A new object **z** will be classified in the class for which the discriminant is the largest. This classification approach produces nonlinear (quadratic) class boundaries, giving the name of the classifier as *quadratic discriminant* rule or Gaussian classifier.

An alternative to this quadratic classifier is to assume that the class covariance matrices Σ*_c_*, *c* = 1,. . .,*K* are all the same. In this case, instead of using a different covariance matrix estimate for each class, a single pooled covariance matrix is used. This can be especially useful when the number of samples per class is low. In this case, calculating a covariance matrix from only a few samples may produce very unreliable estimates. Better results may be obtained by assuming a common variance and using all samples to estimate a single covariance matrix. The resulting classifier uses hyperplanes as class boundaries, hence the name *normal-based linear discriminant*.

To cope with situations when the number of features is comparable with the number of samples, a further simplification can be made to the normal-based linear discriminant, by setting all off-diagonal elements in the covariance matrix to zero. This implies that between-features covariation is disregarded. Such a *diagonal linear discriminant* was found to outperform other types of classifiers on a variety of microarray analyses [16].

The above-presented classifiers work optimally when their underlying assumptions are met, such as the normality assumption. In many cases, some of the assumptions may not be met. However, (as pointed out by one of the anonymous reviewers) what matters in the end for a practical application is how close the estimated class boundaries are to the true class boundaries. This can be assessed through a cross-validation process.

In very recent work, Guo and colleagues [17] have presented a regularized linear discriminant analysis procedure useful when the number of features far exceeds the number of samples.

*k-Nearest neighbor classifier.* The *k*-NN classifier can be seen as a nonparametric method of density estimation [13] and uses no assumption on the data distribution, except for the continuity of the feature variables. The *k*-NN classifier does not require model fitting but simply stores the training dataset with all available vector prototypes of each class. When a new object **z** needs to be classified, the first step in the algorithm is to compute the distance between **z** and all the available objects in the training set, **x** *_i_*, *i* = 1,. . .,*n*. A popular choice of distance metric is the Euclidean distance: 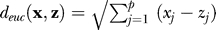 . A thorough discussion of distance functions with application to microarray analysis is given by Gentleman et al. [18].

The distances are ordered and the top *k* training samples (closest to the new object to be predicted) are retained. Let us denote with *n_c_* the number of objects in the training dataset among the *k* ones which belong to the class *c*. The *k*-NN classification rule classifies the new object **z** in the class that maximizes *n_c_*, i.e., the class that is most common among the closest *k* neighbors. The *k*-NN discriminant functions can be written as *g_c_*(**x**) = *n_c_*. When two or more classes are equally represented in the vicinity of the point **z**, the class whose prototypes have the smallest average distance to **z** may be chosen.

*Decision trees.* A special type of classifier is the decision tree [19], which is trained by an iterative selection of individual features that are the most salient at each node in the tree. The input space *X* is repeatedly split into descendant subsets, starting with *X* itself. There are several heuristic methods for constructing decision-tree classifiers. They are usually constructed top-down, beginning at the root node and successively partitioning the feature space. The construction involves three main steps. 1) Selecting a splitting rule for each internal node, i.e., determining the feature together with a threshold that will be used to partition the dataset at each node. 2) Determining which nodes are terminal nodes. This means that for each node we must decide whether to continue splitting or to make the node terminal and assign to it a class label. 3) Assigning class labels to terminal nodes by minimizing the estimated error rate.

**Figure 1:**
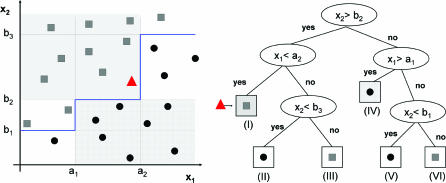
Binary Decision Tree The left panel shows the data for a two-class decision problem, with dimensionality *p* = 2. The points known to belong to classes 1 and 2 are displayed with filled circles and squares, respectively. The decision boundary is shown as the blue thick line in the left panel. The triangle designates a new point, **z**, to be classified. The right panel shows the decision tree derived for this dataset whereas the new point **z** is classified in class 2 (squares). The regions in the input space covered by nodes I and IV in the tree are represented by the dashed areas at the top and bottom of the left panel, respectively.

The most commonly used decision tree classifiers are binary. They use a single feature at each node, resulting in decision boundaries that are parallel to the feature axes (see Figure 1). Although they are intrinsically suboptimal, the resulting classifier is easy to interpret.

**Figure 2:**
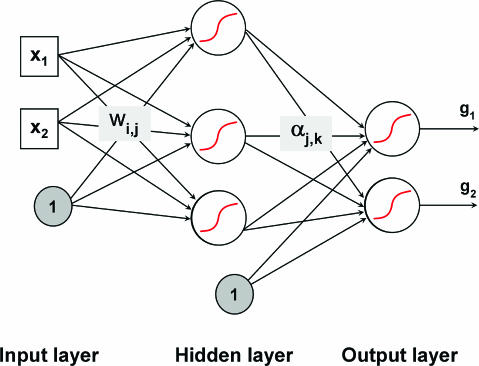
A Schematic Representation of a Feed-Forward Three-Layered Neural Network Two-dimensional data points (*p =* 2) are classified into *K* = 2 known classes. The sigmoid hidden and output units are shown as white circles containing an S-like red curve.

*Neural networks.* The most common neural network architecture used in classification problems is a fully connected, three-layered structure of nodes in which the signals are propagated from the input to the output layer via the hidden layer (see Figure 2). The input layer only feeds the values of the feature vector **x** to the hidden layer. Each hidden unit weights differently all outputs of the input layer, adds a bias term, and transforms the result using a nonlinear function, usually the logistic sigmoid:

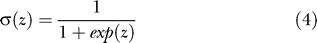



Similarly to the hidden layer, the output layer processes the output of the hidden layer. Usually there is one output unit for each class. The discriminant function implemented by the *k*th output unit of such a neural network can be written as:

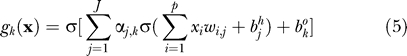



In this equation, *w_i_*_,*j*_ is the weight from the *i*th input unit to the *j*th hidden node, *α_j_*_,*k*_ is the weight from the *j*th hidden unit to the *k*th output node, 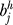 is the bias term of the *j*th hidden unit, 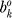 is the bias term of the *k*th output unit. They all represent adjustable parameters and are estimated (learned) during the training process that minimizes a loss function. A commonly used loss function is the sum of squared errors between the predicted and expected signal at the output nodes, given a training dataset.

Consider that *N_T_* training samples are available to train a neural network with *K* output units. The error of the neural network on the training set can be computed as:

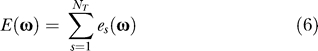
where **ω** represents all the adjustable parameters of the neural network (weights and biases) which are initialized with small random values, and *e_s_* is the error obtained when the *s*th training sample is used as input into the network. The error *e_s_* is defined as proportional to the sum of squared differences between the expected outputs of the network and the actual outputs, given the current values of the weights, i.e.,

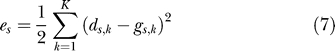



Here, *g_s_*_,*k*_ represents the actual output of the unit *k* for the sample *s*, while *g_s_*_,*k*_ is the desired (target) output value for the same sample. When a sample belongs to the class *k*, it is desired that the output unit *k* fires a value of 1, while all the other output units fire 0. The learning process is done by updating the parameters **ω** such that global error decreases in an iterative process. A popular update rule is the back-propagation rule [20], in which the adjustable parameters **ω** are changed (increased or decreased) toward the direction in which the training error *E*(**ω**) decreases the most.

Equation 6 above can be modified in a way that the training process not only minimizes the sum of squared errors on the training set, but also the sum of squared weights of the network. This *weights regularization* enhances the generalization capability of the model by preventing small variations in the inputs to have excessive impact on the output. The underlying assumption of the weights regularization is that the boundaries between the classes are not sharp.

For more details on theory and practical use of neural networks, please see Duda et al. [12], Ripley [21], Venables and Ripley [22], and references therein.

**Figure 3:**
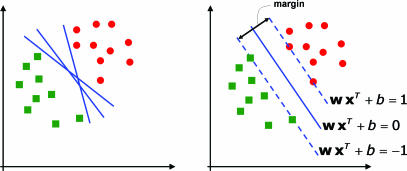
Support Vector Machines Class Boundaries Two-dimensional data points belonging to two different classes (circles and squares) are shown in the left panel. The right panel shows the maximum-margin decision boundary implemented by the SVMs. Samples along the dashed lines are called SVs.

*Support vector machines.* Consider a two-class, linearly separable classification problem, as shown in Figure 3, left panel. While many decision boundaries exist that are capable of separating all the training samples into two classes correctly, a natural question to ask is: are all the decision boundaries equally good? Here the goodness of decision boundaries is to be evaluated as described previously by cross-validation. Among these decision boundaries, SVMs find the one that achieves maximum margin between the two classes. From statistical learning theory, the decision functions derived by maximizing the margin minimize the theoretical upper bound on the expected risk and are thus expected to generalize well [23]. The margin is defined as the distance between a planar decision surface that separates two classes and the closest training samples to the decision surface (see Figure 3, right panel). Let us denote with 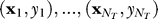 the labeled training dataset where **x** *_i_* ∈ ℜ*^p^*, *y_i_* ∈ {−1,+1}. SVMs find an optimal hyperplane **wx** *^T^* + *b* = 0, where **w** is the *p*-dimensional vector perpendicular to the hyperplane and *b* is the bias. The objective of training SVMs is to find **w** and *b* such that the hyperplane separates the data and maximizes the margin 1 / || **w** ||^2^ (Figure 3, right panel). By introducing non-negative slack variables *ξ_i_* and a penalty function measuring classification errors, the linear SVM problem is formulated as follows:

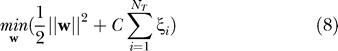
subject to constraints:

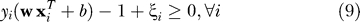
where *C* is a parameter to be set by the user, which controls the penalty to errors. The optimization problem can be reduced to a dual problem with solutions given by solving a quadratic programming problem [23]. The decision function is simply

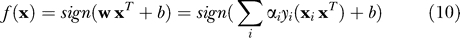
where *α_i_* are coefficients that can be solved through the dual problem. Data points with nonzero *α_i_* are called support vectors (SVs) (e.g., Figure 3, right panel). In SVMs, only SVs contribute to the construction of the decision boundaries.

The linear SVMs can be readily extended to nonlinear SVMs where more sophisticated decision boundaries are needed. This is done by applying a kernel transformation, i.e., simply replacing every matrix product (**x** *_i_* **x** *^T^*) in linear SVMs with a nonlinear kernel function evaluation *K*(**x** *_i_* **x**). This is equivalent to transforming the original input space *X* nonlinearly into a high-dimensional feature space. The training data that are not linearly separable in the original feature space can be linearly separated in the transformed feature space. Consequently, the decision boundaries are linear in the projected high-dimensional feature space and nonlinear in the original input space. Two commonly used kernels include polynomial

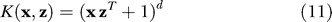
and radial basis function (RBF)

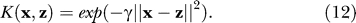



The kernel functions return larger values for arguments that are closer together in feature space.

In constructing linear SVMs for classification, the only parameter to be selected is the penalty parameter *C. C* controls the tradeoff between errors of SVMs on training data and the margin. For nonlinear SVMs, the learning parameters include *C* and parameters associated with the kernels used, e.g., *γ,* in radial basis function (RBF) kernels. In practice, learning parameters are selected through cross-validation methods.

To conclude, the key points with the SVMs are: a) one believes there is a representation of features in which classes can be discriminated by a single hyperplane (perhaps with only a few errors); b) one chooses the hyperplane that lies at the largest distance between sentinel cases near the class boundary (large margin); and c) one can use kernel transformations when data is not linearly separable in the original feature space, but it may be so in the transformed space.

#### Dimensionality reduction.

An important aspect of the classifier design is that in some applications, the dimensionality *p* of the input space is too high to allow a reliable estimation of the classifier's internal parameters with a limited number of samples (*p* ≫ *n*). In such situations, dimensionality reduction may be useful. There are two main categories of approaches to dimensionality reduction. The first one is to obtain a reduced number of new features by combining the existing ones, e.g., by computing a linear combination. *Principal component analysis* (PCA) is one particular method in this branch, in which new variables (principal directions) are identified and may be used instead of the original features. The second type of dimensionality reduction involves *feature selection* that seeks subsets of the original variables that are adequately predictive.

A serious difficulty arises when *p* ≫ *n* is *overfitting*. Most of the procedures examined in this tutorial include a set of tunable parameters. The size of this set increases with *p*. When more tunable parameters are present, very complex relationships present in the sample can often be fit very well, particularly if *n* is small. Generalization error rates in such settings typically far exceed training set error rates. Reduction of the dimensionality of the feature space can help to reduce risks of overfitting. However, automated methods of dimension reduction must be employed with caution. The utility of a feature in a prediction problem may depend upon its relationships with several other features, and simple reduction methods that consider features in isolation may lead to loss of important information.

The statistical pattern recognition literature classifies the approaches to feature selection into *filter methods* and *wrapper methods*. In the former category, a statistical measure (e.g., a *t*-test) of the marginal relevance of the features is used to filter out the features that appear irrelevant using an arbitrary threshold. For instance, marker genes for cancer prediction were chosen based on their correlation with the class distinction and then used as inputs in a classifier [24].

Although fast and easy to implement, such filter methods cannot take into account the joint contribution of the features. Wrapper methods use the accuracy of the resulting classifier to evaluate either each feature independently or multiple features at the same time. For instance, the accuracy of a *k*-NN classifier has been used to guide a genetic algorithm that searched an optimal subset of genes in a high combinatorial space [25]. The main disadvantage of such methods trying to find optimal subsets of features is that they may be computationally demanding. Main advantages of wrapper methods include the ability to: a) identify the most suited features for the classifier that will be used in the end to make the decision, and b) detect eventual synergistic feature effects (joint relevance). More details on feature selection methods and classification can be found in the literature [16,26,27].

## Unsupervised Learning / Cluster Analysis

Clustering is a popular exploratory technique, especially with high dimensionality data such as microarray gene expression [28,29]. This section will introduce the main clustering approaches used with biological data.

### 

#### Overview of clustering algorithms.

Clustering aims at dividing objects into groups (clusters) using measures of similarity, such as one minus correlation or Euclidean distance. For instance, in a microarray experiment the objects can be different tissue samples that can be clustered based on *p*-tuples of gene expression values. Some of the most frequently used clustering techniques include *hierarchical* clustering and *k-means* clustering. Hierarchical clustering creates a hierarchical, tree-like structure of the data. A hierarchical clustering can be constructed using either a bottom-up or a top-down approach. In a bottom-up approach, each data point is initially considered a cluster per se. Subsequently, the clusters are iteratively grouped based on their similarity. In contrast, the top-down approach starts with a unique cluster containing all data points. This initial cluster is iteratively divided into smaller clusters until each cluster contains a single data point. The *k*-means clustering algorithm starts with a predefined number of cluster centers (*k*) specified by the user. Data points are assigned to these centers based on their distance from (similarity to) each center. Subsequently, an iterative process involves recalculating the position of the cluster centers based on the current membership of each cluster and reassigning the points to the *k* clusters. The algorithm continues until the clusters are stable, i.e., until there is no further change in the assignment of the data points.

Another approach to clustering is called *partitioning around medoids* (PAM) [30]. Similarly to *k*-means and hierarchical clustering, PAM starts with computing a dissimilarity matrix (*n* × *n*) from the original data structure (the *n* × *p* matrix of measurements).

Any distance measure can be therefore used in conjunction with PAM. The algorithm maps the resulting distance matrix into a specified number of clusters. The medoids are representations of the cluster centers that are robust with respect to outliers. The robustness is particularly important in the common situation in which many elements do not have a clearcut membership to any specific cluster [31]. A measure of cluster distinctness is the *silhouette* computed for each observation in a dataset, relative to a given partition of the dataset into clusters. The silhouette measure contrasts the average proximity of an observation to other observations in the partition to which it is assigned with the average proximity to observations in the nearest partition to which it is not assigned. This quantity tends to one for a “well-clustered” observation and can be negative if an observation seems to have been assigned to the wrong cluster.

**Figure 4:**
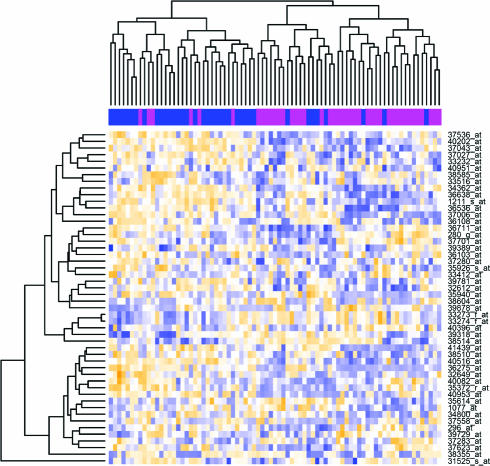
Heat Map of the ALL Data after Filtering Class membership is indicated by a magenta (NEG) or blue (BCR/ABL) stripe at the top of the plot region. Rows correspond to data features (genes), while columns correspond to data points (samples). Hierarchical clustering is applied simultaneously to both rows (genes) and columns (samples) of the expression matrix to organize the display.

In many biological applications, it is desired to cluster both the features and the samples, i.e., both rows and columns of the data matrix X. For instance, with gene expression data one may be interested to cluster both the tissues samples and the genes at the same time. While *k*-means and hierarchical clustering methods can be used, for instance, to group genes that are co-expressed under all measured conditions, they fail to discover local expression patterns, i.e., genes co-expressed across a subset of conditions and independent under other conditions. *Biclustering* methods, on the other hand, allow simultaneous clustering of genes and experimental conditions and uncover local patterns in the data. Given an *n* × *p* matrix, a biclustering algorithm identifies biclusters—a subset of rows that show similar activity patterns across a subset of columns, or vice versa (see Figure 4).

*Self-organizing feature maps* (SOFM) [32,33] are produced by another popular algorithm used in unsupervised applications. Unlike the methods described above, this unsupervised neural network not only finds clusters in the data, but also allows visualization (projection) of the *p*-dimensional data points onto a layer of neurons (usually planar). The neurons are arranged in a rectangular or hexagonal grid and they learn to become prototypes for the training data points. Similar objects will be mapped on the same (or neighboring) neurons, while dissimilar ones will be mapped apart. Thus, the self-organizing feature maps (SOFMs) preserve the intrinsic relationship among the different clusters.

#### Tuning parameters in clustering.

In addition to the type of clustering (e.g., hierarchical, *k*-means, etc.), investigators need to make other choices when employing this technique, including: 1) *distance metric;* and 2) the type of *linkage* (if appropriate). The distance used by the clustering defines the desired notion of similarity between two data points. Distance metrics, i.e., measure of dissimilarity, that are often used, in addition to the *Euclidean* distance (defined in Section 2), are *one minus correlation* distance:

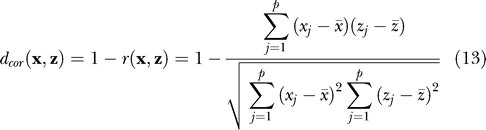
and *Mahalanobis* distance:

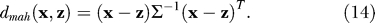



In Equation 14 the covariance matrix Σ can be replaced with the sample estimated covariance matrix defined in Equation 3. Unlike the Euclidian and correlation distances, the Mahalanobis distance allows for situations in which the data may vary more in some directions than in others, and has a mechanism to scale the data so that each feature has the same weight in the distance calculation.

The linkage defines the desired notion of similarity between two groups of measurements. For instance, the *average linkage* uses the mean of the distances between all possible pairs of measurements between the two groups. An extensive discussion of these issues, including the properties of each distance/linkage/clustering algorithm, common pitfalls, and recommendations can be found in Drăghici's monograph [34] and references therein.

## Practicalities Using R

The R language and environment for statistical computing (http://www.r-project.org) is a free open source system with which one can explore a variety of approaches to machine learning. For a comprehensive list of machine learning methods implemented in R, the reader is referred to the CRAN Task View on machine learning (http://cran.r-project.org/src/contrib/Views/MachineLearning.html). In the following description, the bold fixed-width font designates a code segment that can be pasted directly into an R session, while nonbold fixed-width font designates names of packages, or R objects.

The Bioconductor project (http://www.bioconductor.org) includes a software package called MLInterfaces, which aims to simplify the application of machine learning methods to high-throughput biological data such as gene expression microarrays. In this section, we will review some examples that can be carried out by the reader who has an installation of R 2.4.0 or later. First, the CRAN package ctv is installed and loaded. A rich collection of machine learning tools is obtained by executing:

**install.views("MachineLearning")**

The biocLite function is then made available through:

**source("http://www.bioconductor.org/biocLite.R")**

followed by

**biocLite("MLInterfaces")**

which installs a brokering interface to a substantial collection of machine learning functions, tailored to analysis of expression microarray datasets.

### 

#### A leukemia dataset.

After obtaining the biocLite function as described above, the command:

**biocLite("ALL")**

installs a data structure representing samples on 128 individuals with acute lymphocytic leukemia [35]. The following dialogue with R will generate a subset that can be analyzed to understand the transcriptional distinction between B cell ALL cases in which the BCR and ABL genes have fused, and B cell ALL cases in which no such fusion is present:

**library(ALL)**

**data(ALL)**

**# restrict to BCR/ABL or NEG**

**bio = which( ALL$mol.biol %in% c("BCR/ABL", “NEG"))**

**# restrict to B-cell**

**isb = grep("^B", as.character(ALL$BT))**

**bfus = ALL[, intersect(bio,isb)]**

**bfus**

There are 79 samples present, 37 of which present BCR/ABL fusion.

#### Unsupervised methods.

To illustrate simple approaches to unsupervised learning, we will filter the data severely, by focusing on the 50 genes that have the largest variability over all samples as measured by the median absolute deviation. The threshold 1.43 in the next command was determined by checking the data. We then invoke the R heatmap command, with variations on the color scheme, and sample coloring at the top, with magenta bars denoting negative samples (NEG) and blue bars denoting fusion samples (BCR/ABL):

**bfust = bfus[ apply(exprs(bfus),1,mad) > 1.43, ]**

**#get rid of unused levels**

**bfust$mol.biol = factor(bfust$mol.biol)**

**mycols=ifelse(bfust$mol.biol == "NEG",**

**"magenta", "blue")**

**heatmap(exprs(bfust),**

**ColSideColors=mycols,**

**col=cm.colors(256), margins=c(9,9), cexRow=1.3)**

The PAM algorithm can be applied to bfust of class ExpressionSet using the brokering code in the MLInterfaces:

**library(MLInterfaces)**

**dopam = pamB(bfust, k=6)**

**Figure 5:**
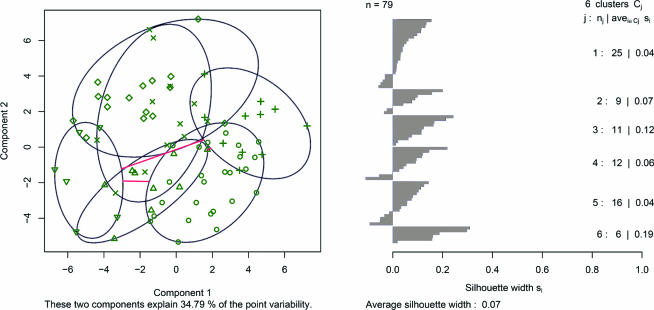
Two Views of the Partition Obtained by PAM Left, PC display; right, silhouette display. The ellipses plotted on the left are cluster-specific minimum volume ellipsoids for the data projected into the PCs plane. These should be regarded as two-dimensional representations of the robust approximate variance–covariance matrix for the projected clusters. The silhouette display comprises a single horizontal segment for each observation, ordered by clusters and by object-specific silhouette value within a cluster. Large average silhouette values for a cluster indicate good separation of most cluster members from members of other clusters; negative silhouette values for objects indicate instances of indecisiveness or error of the given partition.

The graphical output shown in Figure 5 is obtained using the R command:

**plot(RObject(dopam))**

On the left panel of Figure 5, the smallest cluster-specific ellipsoids containing all the data in each cluster are displayed in a two-dimensional principal components (PCs) projection; on the right, the *silhouette* display (see Unsupervised Learning/Cluster Analysis) is presented. High silhouette values indicate “well-clustered” observations, while negative values indicate that an observation might have been assigned to the wrong cluster.

**Figure 6:**
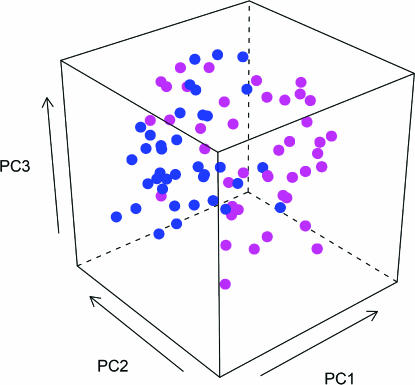
A PCA Plot The 79 samples of the ALL dataset are projected on the first three PCs derived from the 50 original features. The blue and magenta colors are used to denote the known membership of the samples in the two classes, NEG and BCR/ABL, respectively. Note that PCA is an unsupervised data projection method, since the class membership is not required to compute the PCs.

A useful data visualization method, not necessarily related to machine learning, is to project the multidimensional data points onto two or three PCs which are the directions in the feature space showing the largest variability. The R packages pcurve and lattice are used here to compute the PCs and produce a plot of the 79 samples in bfust data (see Figure 6).

**library(lattice);**

**library(pcurve)**

**pc = pca(t(exprs(bfust)))**

**cloud(pc$pcs[,3]∼**

**pc$pcs[,1]+pc$pcs[,2],col=mycols,pch=19,xlab="PC1",**

**ylab="PC2", zlab="PC3")**

#### Supervised methods.

Supervised methods of learning such as trees, neural networks, and SVMs will be illustrated in this section.

The following example uses 50 random samples from bfust data to train a neural network model which is used to predict the class for the remaining 29 samples from bfust. The confusion matrix is computed to assess the classification accuracy. Indices of the training sample are supplied to the trainInd parameter of the nnetB interface of the MLInterfaces package. 

**set.seed(1234) # repeatable random sample/nnet initialization**

**smp = sample(1:79, size = 50)**

**nn1 = nnetB(bfust, “mol.biol", trainInd=smp, size = 5, maxit = 1000,**

**decay =0.01)**

**confuMat(nn1)**

The last line in the code segment above displays the confusion matrix achieved by the neural network classifier on the test samples:

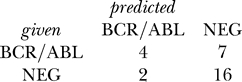



The size parameter in the function nnetB above specifies the number of units in the hidden layer of the neural network, and larger values of the decay parameter impose stronger regularization of the weights. The maxit parameter should be set to a relatively high number to increase the chance that the optimization algorithm converges to a solution. The confusion matrix is computed using the confuMat method on the 29 samples forming the complement of the training set specified by smp. This shows a misclassification rate of 31% = 9/29.

**Figure 7:**
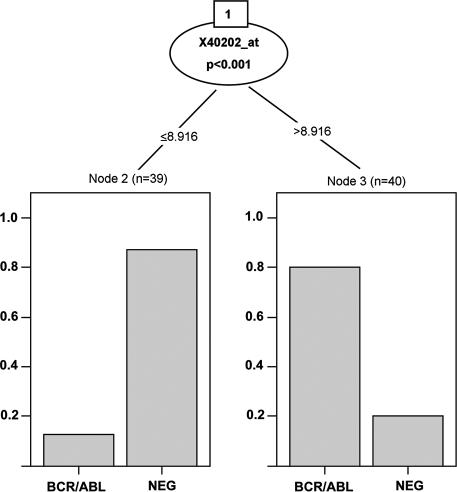
Rendering of a Conditional Tree The figure is obtained with the Ctree function of the party package.

A tree-structured classifier derived from the 50-gene extract from the ALL data is shown in Figure 7. The procedure defines a single split on a single gene (Kruppel-like factor 9), which does a reasonable job of separating the fusion cases—the estimated misclassification rate seems to be about 30%.

**Figure 8:**
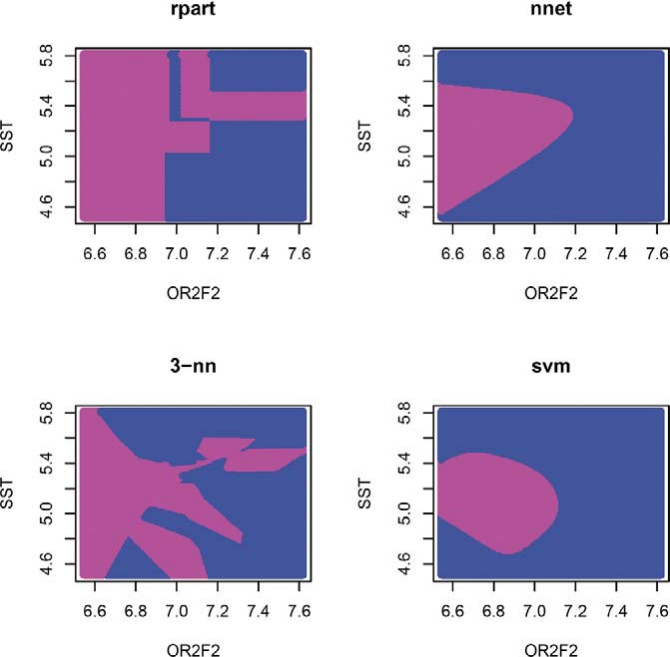
Display of Four Two-Gene Classifiers Top left: CART with minsplit tuning parameter set to 4; top right: a single-layer feed-forward neural network with eight units; bottom left, *k* = 3 nearest neighbors; bottom right, the default SVM from the e1071 package. The planarPlot function of the MLInterfaces package can be used to construct such displays. If the expression level of a given sample falls into the magenta-colored area, then the sample is predicted to have status NEG; if it falls into the blue-colored area, then the sample is predicted to have BCR/ABL status.

Figure 8 depicts the decision regions after learning was carried out with training sets based on two randomly selected genes from ALL data. Qualitative aspects of the decision regions are clear: the tree-structured classifier delivers rectangular decision regions; the neural network fit leads to a smooth, curved decision boundary; the 3-NN fit is very jagged; and the SVM fit is similar to but more compact than the neural net. Of note: considerable interpolation and extrapolation is performed to generate the full decision region representation, and decisions are rendered for feature values for which data are very sparse. Boundaries are sharp, and there is no provision for declaring doubt (although one could be introduced with modest programming for those procedures that do return information on posterior probabilities of class membership.) Last, the fine structure of the regions provided by CART and 3-NN are probably artifacts of overfitting, as opposed to substantively interesting indications of gene interaction.

#### Variable importance displays.

**Figure 9:**
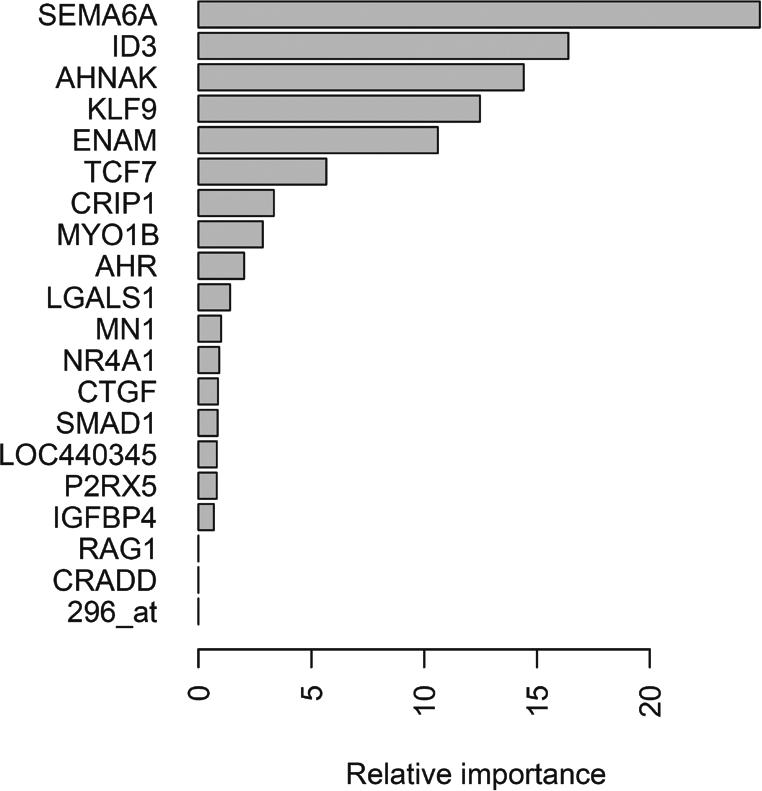
Display of Relative Variable Importance as Computed in a Gradient Boosting Machine Run

Several machine learning procedures include facilities for measuring relative contribution of features in successful classification events. The random forest [36] and boosting [37] methods involve iteration through random samples of variables and cases, and if accuracy degrades when a certain variable is excluded at random from classifier construction, the variable's importance measure is incremented. Code illustrating an application follows, and Figure 9 shows the resulting importance measures. 

**ggg = gbmB(bfust, "mol.biol", 1:50)**

**library(hgu95av2)**

**par(las=2, mar=c(6,9,5,5))**

**plot(getVarImp(ggg), resolveenv=hgu95av2SYMBOL )**

#### Summary.

The R system includes a large number of machine learning methods in easily installed and well-documented packages; the Bioconductor MLInterfaces brokering package simplifies application of these methods to microarray datasets. We have illustrated a number of methods with a demonstration dataset that was obtained by selecting a reduced number of features out of a few tens of thousands that are available in the ALL dataset. The features selected were those varying the most among the samples, regardless of their class membership. While convenient for the purpose of producing Figure 4, the filtering is not theoretically required by any of the unsupervised methods. However, for practical reasons, such as computer memory shortage, most of the implementations of the unsupervised techniques may not work with tens of thousands of features. For the purpose of developing supervised classification models, in addition to these practical limitations, there may not be enough degrees of freedom to estimate the parameters of the models. In such supervised applications, filtering should be used as described in the section Supervised Learning: Dimensionality Reduction. More details on machine learning applications with R can be found in the literature [38].

## Conclusion

Modern biology can benefit from the advancements made in the area of machine learning. Caution should be taken when judging the superiority of some machine learning approaches over other categories of methods. It is argued [39] that the success or failure of machine learning approaches on a given problem is sometimes a matter of the quality indices used to evaluate the results, and these may vary strongly with the expertise of the user. Of special concern with supervised applications is that all steps involved in the classifier design (selection of input variables, model training, etc.) should be cross-validated to obtain an unbiased estimate for classifier accuracy. For instance, selecting the features using all available data and subsequently cross-validating the classifier training will produce an optimistically biased error estimate. Because of inadequate validation schemes, many studies published in the literature as successful have been shown to be overoptimistic [40]. It should be clear from the narrative examples used in this tutorial that choice, tuning, and diagnosis of machine learning applications are far from mechanical. 

## Abbreviations

*k*-NN: *k*-nearest neighbor
PAM: partitioning around medoids
PC: principal component
PCA: principal component analysis
SV: support vector
SVM: support vector machine
**x**: vector
*x*: scalar
X: matrix
*X*: feature space.
